# Correlation between APACHE II score and quality of life among patients discharged from the ICU

**DOI:** 10.1186/cc11011

**Published:** 2012-03-20

**Authors:** L Zubek, L Szabó, L Horváth, A Mesterházi, J Gál, G Élő

**Affiliations:** 1 Semmelweis University, Budapest, Hungary; 2 Markusovszky Hospital, Szombathely, Hungary

## Introduction

The goal of intensive therapy is not only saving the patient's life, but also to restore their quality of life. Based on expected quality of life improvement, a fair allocation of limited available resources can be provided. The assessment scores for the physical state of ICU patients, which correlate with survival, are widely known. However, it would be useful to know if these score systems also correlate with the long-term quality of life. The aim of our study was to investigate the correlation between the APACHE II score and the long-term quality of life after ICU treatment.

## Methods

We have collected data retrospectively from patients treated in our department during the first quarter of 2008. The APACHE II score was calculated for all patients, after which we examined the correlation between this value and the survival of the patients. One year after ICU therapy, the Hungarian version of the EQ-5D questionnaire (measurement consist of five dimensions: mobility, self-care, usual activities, pain/discomfort, anxiety/depression and a visual analog scale about health state) developed by EuroQol Group was sent out by post. The correlation between the APACHE score and quality of life was calculated, the Spearmann rank-order correlation was used.

## Results

During this period, 190 patients were treated in our department. The average of the APACHE II score was 13.23 (±6.99). In total, 25.3% of patients died during treatment; 22.1% died during the first post-treatment year; 27.9% surely survived and 24.7% of patients were unattainable. In our cohort, every patient below 11 points survived and none above 24. The average APACHE score of patients completing the questionnaire was 9.30 (±3.85). They assessed their health as 66% at VAS, although correlation between this value and the APACHE score could not be shown. However, we found statistically significant correlation between the APACHE score and the current mobility of the patients (*P *= 0.021). Based on our data, 34% of the patients had problems with mobility, 36% with usual activity, 62% of patients complained about pain or discomfort, 50% felt anxiety or depression and 18% had problems with self-care.

## Conclusion

ICU admission is associated with a high mortality, a poor physical quality of life and low quality-adjusted life-years for 1 year after discharge. We found that the APACHE II score did not show significant correlation with patient's long-term quality of life, but we detected significant correlation between the APACHE II score and the current mobility of the patients.

